# Using LC and Hierarchical Cluster Analysis as Tools to Distinguish Timbó Collections into Two *Deguelia* Species: A Contribution to Chemotaxonomy

**DOI:** 10.3390/molecules21050569

**Published:** 2016-04-30

**Authors:** Danielle da Costa, Consuelo e Silva, Aline Pinheiro, Débora Frommenwiler, Mara Arruda, Giselle Guilhon, Cláudio Alves, Alberto Arruda, Milton Da Silva

**Affiliations:** Programa de Pós-Graduação em Química, Instituto de Ciências Exatas e Naturais, Universidade Federal do Pará, Campus Universitário do Guamá, 66075-970 Belém-PA, Brazil; danymont2003@yahoo.com.br (D.C.); yumikoyoshioka@yahoo.com.br (C.S.); alinecollares@gmail.com (A.P.); debora.frommenwiler@camag.com (D.F.); mspa@ufpa.br (M.A.); giselle@ufpa.br (G.G.); nahum@ufpa.br (C.A.); arruda@ufpa.br (A.A.)

**Keywords:** *Deguelia*, chemotaxonomy, rotenoids, high-performance liquid chromatography (HPLC), hierarchical cluster analysis (HCA)

## Abstract

The species *Deguelia utilis* and *Deguelia rufescens* var. *urucu*, popularly known as “timbó,” have been used for many years as rotenone sources in insecticide formulations. In this work, a method was developed and validated using a high-performance liquid chromatography-photodiode array (HPLC-PDA) system, and results were analyzed using hierarchical cluster analysis (HCA). By quantifying the major rotenoids of these species, it was possible to establish a linear relation between them. The ratio between the concentrations of rotenone and deguelin for *D. utilis* is approximately 1:0.8, respectively, while for *D. rufescens* var. *urucu* it is 2:1. These results may help to distinguish these species contributing to their taxonomic identification.

## 1. Introduction

The roots of some leguminous species from the *Derris, Lonchocarpus*, *Tephrosia*, and *Deguelia* genera have been extensively used as extracts or even *in situ* in formulations for insecticide, pesticide, and ichthyotoxic purposes [1]. Two Amazonian species have played an important role in the northern region of Brazil: *Deguelia utilis* (A.C.Sm.) A.M.G.Azevedo, whose basionym is *Lonchocarpus* utilis A.C.Sm. and synonym is *Derris nicou* Macbr. (“white timbó”), and *Deguelia rufescens* var. *urucu* (Ducke) A.M.G.Azevedo, whose basionym is *Derris urucu* (Killip & Sm.) J.F. Macbr. and synonym is *Lonchocarpus urucu* Killip & A.C.Sm. (“red timbó”) [2]. Both species have been useful for local indigenous and traditional peoples due to their ichthyotoxic effects [3]. 

Recently, several flavonoids and stilbenes have been isolated from the leaves of *D. utilis* and *D. rufescens* var. *urucu* [4,5]. Although some of these substances have shown antioxidant, neuroprotective, and hypoglycemic activities [4,5,6], it is the roots of these species that are the most used part of these plants in many countries, as pesticides and insecticides, and in applications in the organic agriculture sector [7]. Their biosynthetic markers and active principles are rotenoids, especially rotenone and deguelin, at different levels depending on the species [7,8].

Independent studies developed by Cabras *et al.* [9] and Sherer *et al.* [10] have called into question the safety of rotenone. The first study reported the persistence of rotenone in olive crops, with half-lives of four days; in the second, the authors administered this substance to rats, with results suggesting that it can cause the same symptoms as Parkinson’s disease (PD), resulting in considerable discussion that culminated in the abandonment of rotenone-based pesticides [9,10]. Hancock *et al.* [11] examined the relationship between pesticide exposure and the risk of acquiring PD, comparing insecticide and herbicide classes under different conditions of pesticide application. According to these authors, two specific insecticide classes, organochlorines and organophosphorus compounds, can significantly increase PD risk [11]. It seems like the problem is not exactly the rotenone-based pesticide itself, since synthetic ones showed superior risk for acquiring PD, but exposure to any pesticides during their application, because of the failure to use protective equipment [12]. Isman defends the development and use of known botanical pesticides such as rotenone instead of screening for more plants and bioactives or use of synthetic ones [12], given the numerous advantages in reuptake of botanical pesticides such as the low cost and availability of these plants in tropical countries. The use of rotenone-based plants (*D. rufescens* var. *urucu* and *D. utilis*) as pesticides appears to continue being a viable option especially in developing countries such as Brazil [13,14], if protective equipment is used during application.

Although *D. rufescens* var. *urucu* and *D. utilis* have been widely utilized as inputs for preparing pesticide extracts, there has always been considerable divergence in taxonomic classification of these species as well as difficulty in correctly identifying specimens in the field [2,15]. This difficulty may be related to the similarities between them and to morphological changes brought about by factors such as soil, ultraviolet radiation (UV), humidity, or age of the plant [16]. In the specific case of “white timbó” (*D. utilis*), this difficulty is even greater due to the almost complete lack of flowers as a result of its propagation, having been done using stakes over a long period [17]. 

Knowledge regarding the chemical composition of botanical species has been widely used for quality control of the botanical material and the products obtained from it and for botanical classification, and may contribute in some cases to the relocation of some species to other genera or even different families of plants [18].

The aim of this work is to quantify rotenone and deguelin using a high-performance liquid chromatographic system with a diode array detector (HPLC-PDA) on the roots of *D. rufescens* var. *urucu* and *D. utilis* and, from these data, provide the basis for better differentiation between them using hierarchical cluster analysis (HCA). The results were compared using regression analysis in order to verify the existence of a relation between concentrations of rotenone and deguelin and confirm the identification of these species.

The method used for this purpose, when compared with those described in the literature, can be considered as rapid, with an analysis time of less than ten minutes [19], as simple, because it uses an isocratic mode of elution and does not use acid or buffer solutions [7,19] and cheaper, since it uses a UV detector instead of a mass detector [20]. Although a mass detector is more sensitive than a UV detector, the latter may well be usefully employed in this case, since the rotenoids have excellent chromophoric groups that allow them to be highly detectable. Furthermore, the method developed here takes into account two markers, rotenone and deguelin, instead of one [21], making it possible to establish a relationship between the concentrations of the two rotenoids in question.

## 2. Results

### 2.1. Validation of the HPLC Methodology

#### 2.1.1. Selectivity

**Figure 1:**
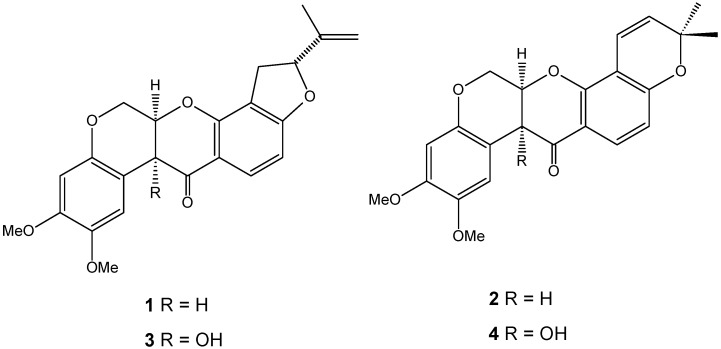
Structures of rotenoids: **1** rotenone, **2** deguelin, **3** rotenolone and **4** tephrosin.

**Figure 2:**
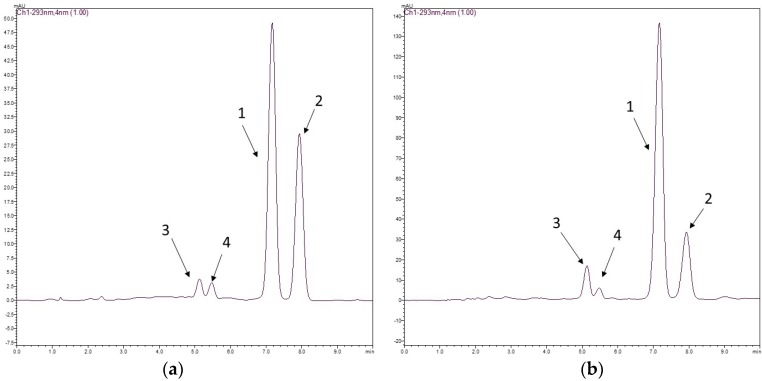
Typical chromatograms of *D. utilis* roots extract (**a**) and *D. rufescens* var. *urucu* roots extract (**b**) for determination of rotenone **1** and deguelin **2**. Chromatographic conditions used: a mixture of acetonitrile/water (60:40; *v*/*v*) as a mobile phase and as stationary phase a Gemini^®^ C18 column (150 × 4.6 mm, 5.0 μm). The injection volume was 20 μL, and the flow rate 1.0 mL min^−1^ at 293 nm.

The chromatographic method had good selectivity, and the chromatograms of the two species displayed two clear peaks without any interference. The average elution times of rotenone (**1**) and deguelin (**2**) were 7.2 and 8.2 min, respectively. Rotenolone (**3**) and tephrosin (**4**) were also observed at 5.2 and 5.6 min, respectively. Figure 1 shows the structures of rotenoids, while Figure 2 shows representative HPLC-PDA chromatograms of the extracts of (a) *D. utilis* roots and (b) *D. rufescens* var. *urucu* roots.

#### 2.1.2. Linearity

The calibration curves were linear within the concentration range assayed. The linearity (*r*^2^), regression equation, the limit of detection (LOD), and the limit of quantitation (LOQ) values of each analyte are shown in Table 1.

#### 2.1.3. Precision and Accuracy

Precision and accuracy were tested using the quality control (QC) solutions and are presented in Table 2.

### 2.2. Determination of Concentrations of Rotenone and Deguelin

After development and validation, the method was applied. All roots samples were submitted to ultrasonic extraction and were injected after filtration (20 μL). Rotenone and deguelin concentrations in ten samples of *D. utilis* (DU-1 to DU-10) and twelve samples of *D. rufescens* var. *urucu* are summarized in Table 3.

### 2.3. Hierarchical Cluster Analysis

**Figure 3:**
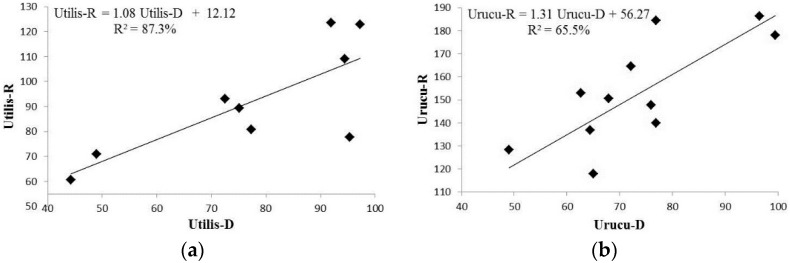
Regression model for both species with respective equation, wherein (**a**) Utilis-R and Utilis-D correspond to rotenone and deguelin concentrations on *D. utilis* roots samples, respectively; and (**b**) Urucu-R refers to rotenone, and Urucu-D to deguelin concentrations on *D. rufescens* var. *urucu* root samples.

The relationship between the concentration of rotenone and deguelin in the species studied can be accessed through the correlation coefficient, and the regression model established for each species is as shown in Figure 3, where we can observe a strong correlation between the concentrations of rotenone and deguelin in *D. rufescens* var. *urucu* (Urucu-R and Urucu-D, respectively), as well as for the same rotenoids in *D. utilis* (Utilis-R and Utilis-D, respectively).

**Figure 4:**
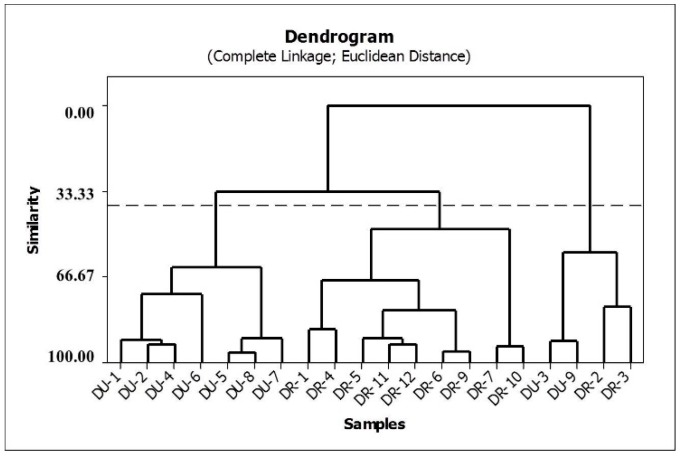
Dendrogram of similarities between production of rotenone and deguelin in the species.

HCA was applied to the auto-scaled data, and the Euclidean distance with the complete linkage method was used to calculate the sample similarities. A hierarchical agglomerative procedure was employed to establish cluster. The results are shown in the dendrogram in Figure 4. In this graph, vertical lines represent roots samples, and horizontal lines represent similarities between samples in terms of the Euclidean distances, which originate from the cluster analysis between pairs of samples, between a sample and a group of samples, and between groups of samples.

## 3. Discussion

These results demonstrate that the production of deguelin increases linearly with rotenone production in accordance with the equation (Utilis-R = 1.08 Utilis-D + 12.12) for *D.* *utilis* (Figure 3a) and (Urucu-R = 1.31 Urucu-D + 56.27) for *D. rufescens* var. *urucu* (Figure 3b). Such equations make it possible to estimate the concentration of one of the rotenoids (rotenone or deguelin) by quantifying the other one.

According to Semmar *et al.* [22], similarities or positive correlations can be interpreted in terms of analogies between chemical structures, synchronic metabolisms, or the co-evolution of two compounds under certain environment conditions. Thus, these positive correlations can translate in terms of metabolism and precursor-product relationships between compounds, while negative correlations can be indicative of competitive processes between two compounds for a common precursor(s), enzyme(s), or substrate(s) [23].

The HCA method classified the 21 valid samples studied into three principal groups. The first group comprises the samples of *D. utilis*, and the second group is composed of the root samples of *D. rufescens* var. *urucu*. The third group is a mix of *D. rufescens* var. *urucu* and *D. utilis* species. Based on this classification, it can be observed that the concentrations of rotenone and deguelin are responsible for the chemical separation between *D. rufescens* var. *urucu* and *D. utilis* species.

The observed linear proportion observed between the concentrations of rotenone and deguelin in the *Deguelia* species studied may be associated with this close biosynthetic relationship between these substances [24].

## 4. Materials and Methods 

### 4.1. General

Compound analytical standards: Rotenone and deguelin were purchased from Sigma-Aldrich, under codes R8875-1G and D0817-5mg, respectively, with >99% and >98% purity, respectively. Acetonitrile (ACN) (HPLC-grade) and ethyl acetate (analytical grade) were purchased from TEDIA (Fairfield, OH, USA). Ultrapure water was obtained from a Millipore (Molsheim, France) Direct-Q_3_^®^ system and was used for all experiments. The ultrasonic bath model was Branson 2510 (Danbury, CO, USA).

### 4.2. Plant Material

The roots of specimens of *D. rufescens* var*. urucu* and *D. utilis* were collected in October 2009 in the EMBRAPA—*Amazônia Oriental* experimental area located at 01° S 48° W, 10 m, situated in the city of Belém, State of Pará, Brazil. The plants were identified and a voucher of each species (exsiccate IAN 181062 and IAN 181063, respectively) was deposited in the IAN Herbarium from Embrapa—*Amazônia Oriental* (Belém, Pará, Brazil). 

### 4.3. Calibration Standards and Quality Control Samples

A stock solution of 1000 μg·mL^−1^ containing the two reference compounds (rotenone and deguelin) was prepared and diluted in acetonitrile to seven appropriate concentrations in order to obtain the working solutions (25; 50; 100; 150; 200; 250; and 300 μg·mL^−1^). The seven calibration standards were prepared by diluting an appropriate aliquot of the working solutions in acetonitrile (2.5; 5.0; 10.0; 15.0; 20.0; 25.0; and 30.0 μg·mL^−1^). Three levels of rotenone and deguelin quality control samples were prepared at the concentrations of 3.0 (low), 12.0 (medium), and 24.0 (high) μg·mL^−1^. 

### 4.4. HPLC Determinations

Analyses were performed using Shimadzu technology (Kyoto, Japan) model LC-20A liquid chromatography fitted with a PDA, SPD-M20A (Shimadzu, Kyoto, Japan). A Phenomenex Gemini^®^ C18 reversed phase analytical column (150 × 4.6 mm I.D., particle size 5.0 μm) was used. The isocratic method was performed for separating rotenone and deguelin using a mobile phase of acetonitrile/water (60:40; *v*/*v*) in 8.5 min. The injection volume was 20 μL, and the flow rate was 1.0 mL·min^−1^. Detection was performed at 293 nm according to satisfactory absorption for both analytes reported in the UV spectrum for rotenone and deguelin. HPLC was controlled and data were elaborated using LC-Solution software (Shimadzu Corporation, Kyoto, Japan, Version 1.25 SP2). 

### 4.5. Validation of Analytical Method

The chromatographic methods were validated to determine the linearity, limit of detection (LOD), limit of quantitation (LOQ), intraday and interday precisions, and accuracies. The precision and accuracy of the analytical method were estimated by intraday and interday analysis of quality control (QC) samples of rotenone and deguelin over a period of three days. QC samples were analyzed in five replicates severally per day to evaluate intraday precision and analyzed over a period of three days for interday precision. During the three days, samples were prepared at high, medium, and low concentrations of five samples each to ensure the analysis was up to standard. The samples were made every day to ensure each batch was new. The precision was calculated by using relative standard deviation (RSD). Accuracy was determined by comparing the concentrations, calculated using calibration curves with known concentrations. The accuracy was expressed as the percentage value of observed concentration to nominal concentration, and the accuracy was expected to be in the range of 85%–115% for each concentration. The RSD should not exceed 15%. The LOD was determined at a signal-to-noise (S/N) ratio of 3, and LOQ was determined at a S/N ratio of 10. 

### 4.6. Preparation of Samples

Samples of 30 cm of roots from 10 specimens of *D*. *utilis* (DU-1 to DU-10) and 12 of *D*. *rufescens* (DR-1 to DR-12) were accurately collected and cleaned and, after drying, were pulverized and homogenized using a mill (Arno, São Paulo, Brazil). The resulting material was stored in glass vials protected from light and humidity at room temperature until extraction. After exhaustive extraction tests, varying solvents (ethanol, ethyl acetate, acetonitrile, and acetone), the volume of solvent, the amount of roots, the time and the repetition of extraction in ultrasonic bath, and the method for extraction of rotenone and deguelin was optimized (with recovery varying between 101.1% to 104.7% for rotenone and from 103.7% to 106.3% for deguelin in the method chosen). OuvirLer foneticamente Dicionário—Ver dicionário detalhado. Extraction of rotenone and deguelin from the material (5 mg) was carried out four times with 3 mL of ethyl acetate with sonication in an ultrasonic bath for 5 min. Samples were evaporated under vacuum, and the dried extract was accurately dissolved in acetonitrile and filtrated on a 0.45-μm nylon membrane. The solutions were placed in an autosampler vial, and the aliquots (20 μL) were injected into the chromatographic system. Three replicates of each sample were analyzed.

### 4.7. Hierarchical Cluster Analysis (HCA)

The databases were submitted to the HCA technique, taking into account the concentration of each rotenoid. The HCA examined the distances between the samples in a data set, and the information was then represented in a two-dimensional plot (dendrogram). The most similar points were grouped forming the clusters, and the process was repeated until all the points were inserted into a unique group [25]. All data were statistically analyzed using MINITAB 14.0 software (Minitab Inc., State College, PA, USA).

### 4.8. Statistical Analysis

Analysis of variance (ANOVA) and regression analysis were performed by MINITAB 14 software (2003). Mean comparisons were performed with Tukey’s test at *p* ≤ 0.05, when appropriate.

## 5. Conclusions

All extracts of *D. rufescens* var. *urucu* presented greater quantities (approximately double) of rotenone when compared to *D. utilis* extracts, making the former species a better source of rotenone for pesticide purposes, since this rotenoid is more active than deguelin.

Quantification results show a linear relation between the ratio of rotenone and deguelin production in both species. The concentration of these rotenoids in *D. utilis* was approximately the same, with deguelin presenting a lower concentration on all samples when compared with rotenone, except for DU-6. This ratio for *D. rufescens* var. *urucu* specimens is about twice more rotenone than deguelin. 

The analytical method developed by HPLC-PDA together with hierarchical cluster analysis can be used as additional tools for differentiating species, making this study a pathway to distinguishing the studied species.

## Figures and Tables

**Table 1:** Linearity (*r*^2^), regression equation, limit of detection (LOD), and limit of quantitation (LOQ) values of rotenone and deguelin.

Analytes	Regression Equation	*r*^2^	LOD ^1^ (μg)	LOQ ^2^ (μg)
Rotenone	y = 43442x − 3906	0.9998	0.01	0.10
Deguelin	y = 23578x − 2709	0.9997	0.02	0.24

**^1^** LOD refers to the limits of detection; **^2^** LOQ refers to the limits of quantitation.

**Table 2:** Intraday and interday precisions and accuracies data.

Analytes	Intraday Precision (RSD%)	Interday Precision (RSD%)	Intraday Accuracy	Interday Accuracy
Rotenone	0.9–12.2	0.9–5.0	93.1–113.8	92.5–107.1
Deguelin	0.8–5.6	1.8–4.8	90.0–107.6	90.0–107.3

**Table 3:** Concentrations of rotenone and deguelin in the species *Deguelia utilis and D. rufescens* var. *urucu*. Values presented in μg of rotenoids per gram of DGR ± CV ^a^.

*D. utilis*			*D. rufescens* var. *Urucu*		
Sample	Rotenone	Deguelin	Sample	Rotenone	Deguelin
DU-1 ^b^	109.08 ± 1.82	94.47 ± 0.75	DR-1 ^c^	184.44 ± 3.58	76.91 ± 3.40
DU-2	122.82 ± 0.37	97.29 ± 0.61	DR-2	117.89 ± 13.62	65.00 ± 8.51
DU-3	70.99 ± 1.40	49.01 ± 1.13	DR-3	128.42 ± 4.11	49.07 ± 1.82
DU-4	123.46 ± 3.30	91.91 ± 3.62	DR-4	164.60 ± 0.71	72.09 ± 1.27
DU-5	93.14 ± 0.06	72.46 ± 0.43	DR-5	136.83 ± 0.43	64.40 ± 0.13
DU-6	77.74 ± 7.23	95.34 ± 5.99	DR-6	147.79 ± 19.22	75.97 ± 10.30
DU-7	80.88 ± 5.96	77.32 ± 3.32	DR-7	178.12 ± 5.95	99.49 ± 1.61
DU-8	89.23 ± 2.84	75.12 ± 0.28	DR-8	278.11 ± 13.44	129.52 ± 3.17
DU-9	60.55 ± 4.19	44.26 ± 2.61	DR-9	140.02 ± 6.92	76.89 ± 2.56
DU-10	ND ^d^	ND	DR-10	186.35 ± 1.69	96.49 ± 2.86
	-	-	DR-11	150.70 ± 15.95	67.93 ± 8.41
	-	-	DR-12	152.87 ± 1.23	62.68 ± 3.90

^a^ DGR: dried and ground roots; ^b^ DU: different samples of *D. utilis*; ^c^ DR: different samples of *D. rufescens* var. *urucu*; ^d^ ND: rotenone and deguelin were not detected in these samples.

## Abbreviations

The following abbreviations are used in this manuscript:

HPLC	high-performance liquid chromatography
PDA	photodiode array
HCA	hierarchical cluster analysis
PD	Parkinson’s disease
UV	Ultraviolet
LOD	limit of detection
LOQ	limit of quantitation
QC	quality control
DR	*Deguelia rufescens* var. *urucu*
DU	*Deguelia utilis*
ND	not detected
RSD	relative standard deviation
